# Keep it simple: vascular risk factors and focal exam findings correctly identify posterior circulation ischemia in “dizzy” patients

**DOI:** 10.1186/s12873-016-0101-6

**Published:** 2016-09-13

**Authors:** Karen Chen, Andrea L. C. Schneider, Rafael H. Llinas, Elisabeth B. Marsh

**Affiliations:** The Johns Hopkins University School of Medicine, 600 North Wolfe St. Phipps 446C, Baltimore, 21287 MD USA

**Keywords:** Dizziness, Triage, Stroke, CT angiogram

## Abstract

**Background:**

Dizziness is a common chief complaint of patients presenting to the Emergency Department (ED). Physicians must quickly and accurately identify patients whose etiology is most likely ischemia. Additional tools are available, but often require further training (vestibular testing) or are costly and not always readily available (magnetic resonance imaging (MRI)). This study evaluates the ability of a routine history and simple physical examination to correctly identify dizzy patients with posterior circulation ischemia, and the added utility of CT angiography (CTA).

**Methods:**

We performed a retrospective analysis of all individuals presenting to the ED with a reported chief complaint of dizziness. Neurology was consulted and CTA ordered at the discretion of the ED provider. Demographic, medical, and radiographic variables were evaluated along with final diagnosis. Multivariable logistic regression and ROC analysis were used to determine factors associated with ischemia, the sensitivity of vascular risk factors and focal exam findings in predicting ischemia, and the additional benefit, if any, of CTA.

**Results:**

One thousand two-hundred sixteen individuals meeting inclusion criteria presented to the ED over a 2 year period and were included in analysis. One hundred (8.2 %) were diagnosed with posterior circulation ischemia. For the entire cohort, age (OR 1.4 per 10 years, *p* < 0.0001), systolic blood pressure (OR 1.3 per 10 mmHg, *p* < 0.0001), and focal exam findings (OR 28.69, *p* < 0.0001) were most significantly associated with ischemia in multivariable modeling. When age, race, sex, presence of vascular risk factors, and focal neurologic findings were entered into ROC analysis, the AUC for correctly identifying posterior circulation ischemia was 0.90. In the subset of patients who underwent CTA (*n* = 87), the AUC did not improve (0.78 with and without CTA in ROC analysis, *p* = 0.52).

**Conclusions:**

A vascular risk assessment and neurological examination are adequate for risk stratification of ischemia in the dizzy patient and should remain the standard evaluation.

## Background

Dizziness is one of the most common complaints of individuals presenting to the Emergency Department (ED). In the United States alone, there are approximately 7.5 million cases each year [1]. Subjective “dizziness” can be reported in a variety of conditions ranging from dehydration, pregnancy, and benign paroxysmal positional vertigo, to cardiac arrhythmia and acute ischemic stroke of the posterior circulation [2]. While the cause of dizziness is often straightforward and most commonly found to be benign [1], it is critical for ED physicians to be able to quickly and accurately differentiate patients who require further diagnostic work-up for more serious causes such as ischemia rather than solely symptomatic management [3].

Evaluation of dizziness is typically based on clinical suspicion and available resources. Currently, no clear practice guidelines exist [4, 5]. Prior studies suggest that age and neurologic deficits [6–8], along with vascular risk factors [6, 9], are associated with increased likelihood that dizziness is due to ischemia; and the consensus of expert opinion is that history and physical examination are most appropriate for initial evaluation [10]. However, there has been variability across studies in the factors evaluated within the clinical history [11] and physical exam [12], making creation of a useful model for risk stratification difficult. Most notably, the examination can take many forms, requiring varying degrees of training and expertise. Most ED providers are comfortable with a basic neurological assessment looking for signs of weakness, double vision, slurred speech, and sensory loss; however, far fewer have experience with more extensive vestibular testing (e.g., classification of nystagmus, assessment of saccades, head impulse testing, Dix Hallpike maneuver) [13].

Along with the physical examination, neuroimaging can be a useful tool for risk stratification in those for whom the diagnosis is not immediately clear; though a noncontrast head CT has been shown to be low yield in prior studies [14, 15]. Magnetic resonance imaging (MRI) is the gold standard to detect acute infarct [16], but is costly and not readily available at all medical institutions [17]. Given the prevalence of dizziness in the ED, the routine use of MRI as a screening tool is prohibitive. Computed tomography angiogram (CTA) is both more available and cost efficient, rendering it an attractive alternative [17]. CTA can be used to not only to determine the patency of the vasculature, but to assess the burden of underlying vascular disease. The presence of calcification within coronary vessels on cardiac CT has been used to gauge risk for myocardial infarction [18]; however, the role of CTA as a predictor of stroke has not been well explored [19]. Given that CTA is not an entirely benign procedure, requiring the use of radiation and iodinated contrast [20], it would be useful to better understand the added utility, if any, of CTA in accurately identifying individuals who are at highest risk for ischemia.

This study was performed to determine the ability of a standard vascular risk factor assessment and simple neurologic screen to identify patients with dizziness secondary to ischemia, and subsequently the added utility of CTA for risk stratification in individuals with diagnostic uncertainty. Results may lead not only to improved risk assessment, but a significant savings in healthcare costs.

## Methods

### Study population

This study was approved by the Johns Hopkins University School of Medicine Institutional Review Board. A retrospective chart review was performed for all adults (greater than or equal to 18 years of age) presenting to the Johns Hopkins Bayview Medical Center ED, an urban tertiary referral center, from January 2012 through December 2013 whose chief complaint within the electronic medical record (EMR) on presentation to the ED was: dizziness, vertigo, or imbalance. The EMR was further reviewed to ensure accuracy of the search results, and data were collected regarding patient demographics (age, race, sex); medical variables (admission systolic and diastolic blood pressure (mmHg), use of antiplatelet and/or anticoagulant medications, history (by patient report) of: diabetes mellitus, coronary artery disease (CAD), hypertension, hyperlipidemia, and tobacco use); physical exam findings (double vision, dysarthria, dysphagia, facial droop, unilateral motor and/or sensory symptoms); laboratory tests (white blood cell count (WBC), hematocrit; erythrocyte sedimentation rate (ESR), glycosylated hemoglobin (HgbA1c), troponin, low-density lipoprotein (LDL), international normalized ratio (INR), serum sodium, blood urea nitrogen, creatinine, glucose); neuroimaging (brain CT, MRI); and final diagnosis. The cause of dizziness was determined by review of the completed work-up (including additional neuroimaging) and discharge summary. The primary outcome of interest was posterior circulation ischemia (stroke or transient ischemic attack (TIA)). Neurology was consulted at the discretion of the ED physician. Stroke was adjudicated from the EMR according to discharge diagnosis and confirmed with neuroimaging results. Posterior circulation stroke was defined by a hyperintensity on diffusion-weighted MRI or hypodensity on head CT located in the vascular territories fed by the vertebrobasilar system (brainstem, cerebellum, occipital lobe, and thalamus supplied by the posterior cerebral artery) that corresponded to presenting symptoms. TIA was determined by the primary neurology team caring for the patient (discharge diagnosis) and defined as a transient deficit most likely due to an underlying vascular cause without lasting evidence on neuroimaging. No intracerebral hemorrhages were included. Charts and neuroimaging were reviewed independently by two separate reviewers with 20 % overlap of cases.

### Neuroimaging

A subset of patients underwent CTA as part of their diagnostic work-up based on the discretion of the treating ED and Neurology teams. The characteristics of this subgroup are detailed in our Results Section and compared to the remainder of the cohort. CTAs were retrospectively reviewed by a board certified vascular neurologist for the presence of calcium within the cervical and/or intracranial vessels, evidenced by an area of hyperdensity with Hounsfield units consistent with bone/calcium (HU ≥1000). The presence of calcium within any vascular bed was recorded as well as presence in specific vascular beds: proximal vertebral artery, distal vertebral artery, basilar artery, proximal carotid artery, distal carotid artery, and aortic arch.

### Statistical analysis

Analyses were performed using Stata SE version 13 (College Station, Texas). Univariate analyses were performed using Student’s paired *t*-tests (for continuous variables) and chi square tests/Fisher’s exact tests (for categorical variables). The primary objective was to determine factors associated with posterior circulation ischemia in patients presenting to the Emergency Department with dizziness and the ability of a basic vascular risk assessment and neurologic screen to identify individuals with posterior circulation ischemia. A secondary aim was to determine the added utility of CTA for those patients thought to require additional imaging. For the primary analysis, covariates significant in univariate analysis (*p* < 0.05), or those thought a priori to be of potential clinical significance, were entered into a multivariable logistic regression analysis of the entire population, with posterior circulation ischemia (stroke or TIA) as the dependent variable. To determine the added benefit of CTA, subsequent models were generated including only patients who underwent CTA. A receiver operating characteristic (ROC) analysis was performed comparing the area under the curve (AUC) using vascular risk factors and clinical exam with and without inclusion of CTA findings, along with sensitivity/specificity analyses.

## Results

### Final included cohort

One thousand two hundred sixty-nine patients aged 18 years or greater were identified by their electronic medical record as potential subjects. Patients were excluded from further analysis if they left the ED without evaluation/laboratory tests (*n* = 53), leaving 1,216 patients for final analysis. Eighty-seven underwent CTA as part of their work-up and were included in the secondary analysis. Inter-rater agreement was 99 % for review of both the EMR and neuroimaging results.

### Factors associated with posterior circulation ischemia

The overall age of the cohort was 56 years (+/−19). Forty-two percent were male and 31 % were black. The average systolic blood pressure was 137 mmHg (+/−26). Sixteen percent of the cohort had coronary artery disease; 63 % hypertension; 26 % diabetes; 41 % hyperlipidemia; and 49 % were previous or active smokers. Fifteen percent were noted to have double vision; 8 % dysarthria; 8 % a facial droop; and 18 % unilateral motor/sensory deficits. One hundred (8.2 %) were diagnosed with posterior circulation ischemia (stroke = 71 or TIA = 29). Forty-five percent of strokes were located in the pons, 30 % the cerebellum, 13 % occipital lobe or thalamus (PCA territory), 8 % medulla, and 4 % midbrain. The most common other discharge diagnoses included: dehydration/orthostatic hypotension, arrhythmia, anemia, hyper/hypoglycemia, and systemic illness such as myocardial infarction or GI illness. Only five percent of the non-ischemic diagnoses were neurological (*n* = 66): 71 % benign paroxysmal positional vertigo/vestibular neuritis, 18 % vestibular migraine, and 11 % vertigo.

#### Univariate analysis (entire cohort)

Male sex (*p* = 0.005), older age (*p* < 0.0001), and elevated systolic blood pressure (*p* < 0.0001) were associated with posterior circulation ischemia in univariate analysis (Table 1). Ischemic patients were more likely to have the following vascular risk factors: diabetes (*p* = 0.062), hypertension (*p* = 0.001), hyperlipidemia (*p* = 0.002), and history of smoking (*p* = 0.004). The presence of focal neurologic deficits including: dysarthria (*p* < 0.0001), facial droop (*p* < 0.0001), and unilateral weakness or numbness (*p* < 0.0001) was also associated with ischemia.

**Table 1:** Patient characteristics. Univariate analysis for the cohort stratified by posterior circulation TIA/stroke status

Variable	Total cohort (*n* = 1216)	Non-stroke (*n* = 1116)	Posterior circulation TIA/Stroke (*n* = 100)	*p*-value
Age (mean years (SD))	56 (19)	55 (19)	67 (15)	<0.0001
Sex (n (% male))	508 (42 %)	453 (41 %)	55 (55 %)	0.005
Race (n (% black))	374 (31 %)	351 (31 %)	23 (23 %)	0.079
Systolic blood pressure (mean mmHg (SD))	137 (26)	135 (25)	161 (26)	<0.0001
Vascular Risk Factors (n (%))				
Coronary artery disease	198 (16 %)	178 (16 %)	20 (20 %)	0.293
Diabetes	318 (26 %)	284 (25 %)	34 (34 %)	0.062
Hypertension	769 (63 %)	691 (62 %)	78 (78 %)	0.001
Hyperlipidemia	495 (41 %)	440 (39 %)	55 (55 %)	0.002
History of smoking	608 (49 %)	545 (49 %)	63 (63 %)	0.004
Neurologic Deficits (n (%))				
Dysarthria	56 (5 %)	14 (1 %)	42 (42 %)	<0.0001
Diplopia	108 (9 %)	91 (8 %)	17 (17 %)	0.624
Facial droop	56 (5 %)	11 (<1 %)	56 (56 %)	<0.0001
Unilateral symptoms^a^	126 (10 %)	56 (5 %)	70 (70 %)	<0.0001

^a^one-sided motor weakness or sensory abnormality

#### Multivariable modeling

Based on univariate results, age, sex, blood pressure, the presence of any vascular risk factor, and any focal neurologic exam finding (double vision, dysarthria, dysphagia, or unilateral motor/sensory complaints) were entered into multivariable regression analysis. Race was also included given the high suspicion it may also be clinically relevant. Individual risk factors and exam findings were condensed into single variables for simplification and to decrease the number of variables within the model. Advanced age (OR 1.4 per 10 years, *p* < 0.0001), systolic blood pressure (OR 1.3 per 10 mmHg, *p* < 0.0001), and focal exam findings (OR 28.69, *p* < 0.0001) remained significantly associated with posterior circulation ischemia in the overall population in multivariable models (Table 2).

**Table 2:** Multivariable model of factors associated with ischemia

Variable	Odds ratio	95 % Confidence interval
Age (per 10 years)	1.3	1.1–1.5
Sex (male)	1.5	0.9–2.6
Race (black)	0.8	0.4–1.4
Systolic Blood Pressure (per 10 mmHg)	1.3	1.1–1.4
Presence of vascular risk factor	1.2	0.4–3.7
Presence of focal deficit	28.7	16.0–51.4

### CTA subgroup

Eighty-seven patients underwent CTA for additional risk assessment. These patients were more likely to be older, white, male, and smokers; with neck pain; focal findings on exam; and higher blood pressures, glucose, HbA1c and LDL values (all *p* < 0.05). A history of diabetes, hypertension, and CAD alone were not significantly associated with patients undergoing further risk assessment with CTA. Overall results for factors associated with ischemia were similar within the subgroup who underwent CTA (*n* = 87) (Table 3), though only focal exam findings remained significant in multivariate analysis for this group (OR 5.38, *p* = 0.003). “Any calcium” on CTA was not a significant predictor of ischemia when the model was fully-adjusted (*p* = 0.38). Only the presence of calcium within the distal internal carotid artery (ICA) was associated with ischemia (OR 3.64, *p* = 0.036) in multivariable modeling.

**Table 3:** Univariate analysis for those undergoing CTA

Variable	Non-stroke (*n* = 36)	Posterior circulation TIA/Stroke (*n* = 51)	*p*-value
Age (mean years (SD))	61 (16)	65 (15)	0.178
Sex (n (% male))	16 (44 %)	34 (67 %)	0.039
Race (n (% black))	4 (11 %)	11 (22 %)	0.203
Systolic blood pressure (mean mmHg (SD))	149 (24)	158 (22)	0.093
Vascular Risk Factors (n (%))			
Coronary artery disease	6 (17 %)	12 (24 %)	0.436
Diabetes	8 (22 %)	15 (29 %)	0.454
Hypertension	24 (67 %)	35 (69 %)	0.847
Hyperlipidemia	17 (47 %)	28 (55 %)	0.480
History of smoking	28 (50 %)	32 (63 %)	0.371
Neurologic Deficits (n (%))			
Dysarthria	2 (5 %)	21 (41 %)	<0.0001
Diplopia	8 (22 %)	10 (20 %)	0.665
Facial droop	0 (<1 %)	23 (45 %)	<0.0001
Unilateral symptoms^a^	8 (22 %)	34 (67 %)	<0.0001
Any calcium on CTA (n)	24 (67 %)	36 (71 %)	0.697
Specific distribution of calcium on CTA (n)			
Proximal vertebral artery	5 (14 %)	8 (16 %)	0.817
Distal vertebral artery	2 (6 %)	5 (10 %)	0.473
Basilar artery	0 (<1 %)	3 (6 %)	0.139
Proximal carotid artery	14 (39 %)	25 (49 %)	0.349
Distal carotid artery	10 (28 %)	30 (59 %)	0.004
Aortic arch	15 (42 %)	26 (51 %)	0.391

^a^one-sided motor weakness or sensory abnormality

### ROC analysis: utility of cta in addition to history and exam in predicting ischemia

**Fig. 1:**
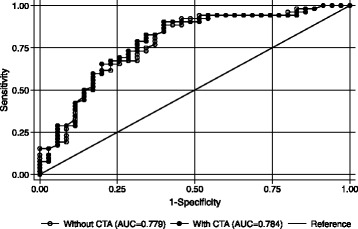
The model was no better at identifying posterior circulation ischemia (identical AUC) when CTA findings were added

We next evaluated how well factors significant in univariate analysis identified patients with posterior circulation ischemia over benign causes of dizziness. Age, race, sex, the presence of any vascular risk factor, and any focal neurologic deficit were entered into the model for ROC analysis for the entire population. The area under the curve (AUC) predicting ischemic stroke was 0.90. The positive predictive value of the model was 57 % and the negative predictive value was 94 %. To evaluate the added benefit of CTA in predicting ischemia, we looked specifically at the cohort who underwent additional neuroimaging (*n* = 87). The AUC for these same variables without adding CTA to the model was 0.78. The AUC did not change when any calcium on CTA (AUC = 0.78) or calcification of the distal ICA (AUC = 0.78) was added to the model (p-value for comparison with and without inclusion of any CTA findings = 0.52, Fig. 1).

## Discussion

Our study suggests that in patients presenting to the ED with subjective dizziness, vascular risk factors and positive focal exam findings on a basic neurological assessment are able to correctly identify the majority of patients with posterior circulation ischemia. Additionally, it suggests that the absence of these factors is reassuring against ischemia. Furthermore, while calcification on CTA is associated with posterior circulation ischemia, it does not significantly increase the ability to correctly identify those at highest risk.

The association between ischemia and vascular risk factors is not surprising and is consistent with prior studies showing that patients with a history of hypertension, CAD, smoking, and/or hyperlipidemia are more likely to be at risk for transient ischemic attack or stroke [1, 21]. Previous studies have also demonstrated the association between ischemia and focal exam findings such as facial droop [22] or eye movement abnormalities [23]. Importantly, we demonstrate that relatively basic examination findings (e.g., weakness and sensory findings) are predictive of ischemia without the additional need for expertise in vestibular testing. The positive predictive value of our model was relatively good at 57 %. While additional examination findings (e.g., direction changing nystagmus, negative head impulse test) may increase the sensitivity, in some cases more so than acute MRI [23], in the majority of cases they are not required for an accurate risk assessment. Furthermore, the negative predictive value of our model was 94 %, indicating that an absence of these variables is reassuring against the presence of ischemia, similar to other screening tests with negative predictive values in the mid to high 90th percentiles [24]. Given the importance of neurologic findings in our model, we evaluated the negative predictive value of examination findings alone. At 92 %, it was lower than that of our full model, indicating that also considering basic demographic information and vascular risk factors was helpful in risk stratification.

Factors associated with ischemia were similar within the group who underwent CTA when compared to the entire cohort, though the significance of vascular risk factors was not as strong in the former group. This phenomenon may be due to a smaller sample size, or that individuals were chosen for CTA based on the fact that they were determined by ED physicians to be higher risk based on risk assessment despite diagnostic uncertainty. Our results suggest that CTA does not further improve risk stratification in this group. Prior research has also suggested that the more frequent use of neuroimaging for patients presenting with dizziness does not significantly strengthen ED physicians’ clinical acumen [25]. However, there has been no prior data in the literature on the specific utility of CTA to differentiate patients whose subjective dizziness is caused by ischemia versus other etiologies. In our cohort, a “positive CTA” did not significantly increase the AUC predicting ischemia, arguing against exposure to radiation and contrast for the majority of individuals. This is not to say that these tools have no place in the work-up; CTA can be extremely helpful in evaluating the patency of the cerebral vasculature, however, it does not significantly improve risk stratification over clinical suspicion determined by history and physical exam.

Though it did not significantly improve the ability to identify patients with dizziness due to ischemia, we did find that the presence of any calcification on CTA is associated with ischemia, particularly in the distribution of the distal ICA. This finding is interesting, and it is most likely due to the fact that the distal ICA is one of the most common locations for calcification. The presence of calcium in this location may merely be another marker for vascular disease. It is notable that the proximal ICA had relatively high rates of calcification in both those with ischemic stroke and those without, which may indicate that the distal carotid may be a better marker of more advanced vascular disease. Alternatively, it is possible that calcium within the intracranial vasculature has a higher predictive value specifically for stroke compared to calcium present at other locations, or that our relatively small sample size of patients undergoing CTA resulted in insufficient power to detect a significant difference with respect to location, and a larger study may have power to show that calcification within a specific vascular bed is a better predictor of stroke within that territory.

Our study is not without limitations. It includes a relatively small number of patients from a single institution, particularly with respect to the number of patients who underwent CTA. Additionally, the true number of strokes and TIAs is unknown. Finally, given the observational retrospective nature of the study, patients were not randomized to CTA and instead underwent testing based on the discretion of the treating teams whose practices may differ from that of other institutions. However, we believe our finding that vascular risk factors and the clinical examination alone are able to identify individuals with posterior circulation ischemia is both quite strong and generalizable; and that, at least for our patient population, the model’s ability to identify patients with ischemia does not improve for those who, for whatever reason (diagnostic uncertainty, perception of “high risk”), are chosen to undergo additional imaging. Our study is consistent with other published literature [6, 9, 26] and highly suggests that the conventional use of stroke risk factors and findings on physical examination to identify ischemia in patients with subjective dizziness is sufficient in the majority of cases to determine appropriate work-up and management without the use of more sophisticated examination techniques, specialized training in vestibular testing, or further neuroimaging.

## Conclusions

In patients presenting to the ED with subjective dizziness, performing a careful history of vascular risk factors and basic neurological assessment looking for focal deficits (dysarthria, diplopia, and unilateral motor/sensory symptoms) will correctly identify most individuals presenting with dizziness secondary to ischemia. In addition, the absence of these findings indicates the likelihood of ischemia is probably low. The presence of calcification within the cervical and intracranial vessels on CTA can be associated with posterior circulation ischemia, but in our population did not add significant utility above clinical acumen. A vascular risk assessment and neurological examination are adequate for risk stratification of ischemia in the dizzy patient and should remain the standard evaluation.

## Abbreviations

AUC: Area under the curve
CAD: Coronary artery disease
CT(A): Computerized tomography (angiogram)
ED: Emergency department
EMR: Electronic medical record
ESR: Erythrocyte sedimentation rate
HU: Hounsfield units
ICA: Internal carotid artery
INR: International normalized ratio
LDL: Low density lipoprotein
MRI: Magnetic resonance imaging
OR: Odds ratio
PCA: Posterior cerebral artery
ROC: Receiver operating characteristic curve
TIA: Transient ischemic attack
WBC: White blood cell count
